# Diaqua­bis­(2-hy­droxy­benzoato-κ*O* ^1^)bis­(nicotinamide-κ*N* ^1^)cadmium–diaqua­bis­(2-hy­droxy­benzoato-κ^2^ *O* ^1^,*O* ^1′^)(nico­tin­amide-κ*N*)cadmium–water (1/2/4)

**DOI:** 10.1107/S1600536813006168

**Published:** 2013-03-09

**Authors:** Nagihan Çaylak Delibaş, Hacali Necefoğlu, Tuncer Hökelek

**Affiliations:** a Department of Physics, Sakarya University, 54187 Esentepe, Sakarya, Turkey; b Kafkas University, Department of Chemistry, 63100 Kars, Turkey; c Hacettepe University, Department of Physics, 06800 Beytepe, Ankara, Turkey

## Abstract

The crystal structure of the title compound, [Cd(C_7_H_5_O_3_)_2_(C_6_H_6_NO)_2_(H_2_O)_2_]·2[Cd(C_7_H_5_O_3_)_2_(C_6_H_6_NO)(H_2_O)_2_]·4H_2_O, consists of two kinds of Cd^II^ complexes (*A* and *B*) and lattice water mol­ecules. In complex *A*, [Cd(C_7_H_5_O_3_)_2_(C_6_H_6_NO)_2_(H_2_O)_2_], the Cd^II^ cation is located on an inversion center and is coordinated by two salicylate anions, two nicotinamide (NA) ligands and two water mol­ecules in a slightly distorted octa­hedral geometry. In complex *B*, [Cd(C_7_H_5_O_3_)_2_(C_6_H_6_NO)(H_2_O)_2_], the Cd^II^ cation is coordinated by two salicylate anions, one nicotinamide (NA) ligand and two water mol­ecules in an irregular seven-coordinate geometry. There are extensive intra­molecular O—H⋯O and weak C—H⋯O hydrogen bonds as well as extensive inter­molecular O—H⋯O and N—H⋯O hydrogen bonding in the crystal structure. π–π stacking between the pyridine and benzene rings, between the benzene rings, between the benzene and pyridine rings and between the pyridine rings [centroid–centroid distances = 3.5989 (10), 3.6005 (10), 3.5800 (9) and 3.5205 (10) Å, respectively] further stabilize the crystal structure. A weak N—H⋯π inter­action also occurs. One of the lattice water mol­ecules is disordered over two positions with an occupancy ratio of 0.70:0.30.

## Related literature

For related structures, see: Greenaway *et al.* (1984 ▶); Hökelek & Necefoğlu (1996 ▶); Hökelek *et al.* (2009*a* ▶,*b* ▶,*c* ▶,*d* ▶).
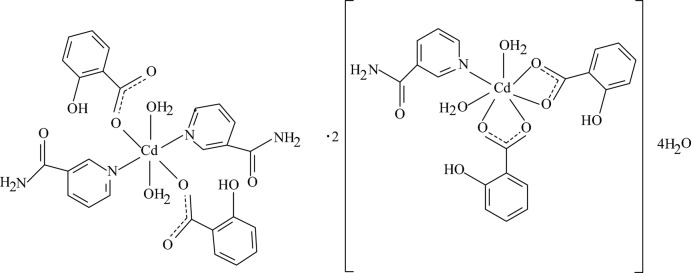


## Experimental

### 

#### Crystal data

[Cd(C_7_H_5_O_3_)_2_(C_6_H_6_NO)_2_(H_2_O)_2_]·2[Cd(C_7_H_5_O_3_)_2_(C_6_H_6_NO)(H_2_O)_2_]·4H_2_O
*M* *_r_* = 1828.56
Triclinic, 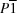

*a* = 10.3446 (2) Å
*b* = 13.5779 (3) Å
*c* = 14.6586 (3) Å
α = 71.226 (3)°
β = 71.364 (3)°
γ = 69.221 (2)°
*V* = 1772.85 (7) Å^3^
*Z* = 1
Mo *K*α radiation
μ = 0.99 mm^−1^
*T* = 100 K
0.42 × 0.32 × 0.29 mm

#### Data collection

Bruker Kappa APEXII CCD area-detector diffractometer
Absorption correction: multi-scan (*SADABS*; Bruker, 2005 ▶) *T*_min_ = 0.691, *T*_max_ = 0.751
31862 measured reflections
8814 independent reflections
8335 reflections with *I* > 2σ(*I*)
*R*_int_ = 0.019

#### Refinement

*R*[*F* ^2^ > 2σ(*F* ^2^)] = 0.019
*wR*(*F* ^2^) = 0.050
*S* = 1.07
8814 reflections
545 parameters
12 restraints
H atoms treated by a mixture of independent and constrained refinement
Δρ_max_ = 0.78 e Å^−3^
Δρ_min_ = −0.55 e Å^−3^

### 

Data collection: *APEX2* (Bruker, 2007 ▶); cell refinement: *SAINT* (Bruker, 2007 ▶); data reduction: *SAINT*; program(s) used to solve structure: *SHELXS97* (Sheldrick, 2008 ▶); program(s) used to refine structure: *SHELXL97* (Sheldrick, 2008 ▶); molecular graphics: *ORTEP-3 for Windows* (Farrugia, 2012 ▶); software used to prepare material for publication: *WinGX* (Farrugia, 2012 ▶) and *PLATON* (Spek, 2009 ▶).

## Supplementary Material

Crystal structure: contains datablock(s) I, global. DOI: 10.1107/S1600536813006168/xu5682sup1.cif

Structure factors: contains datablock(s) I. DOI: 10.1107/S1600536813006168/xu5682Isup2.hkl

Additional supplementary materials:  crystallographic information; 3D view; checkCIF report

## Figures and Tables

**Table 1:** Selected bond lengths (Å)

Cd1—O2	2.3279 (11)
Cd1—O5	2.3200 (12)
Cd1—N1	2.3118 (13)
Cd2—O6	2.5814 (13)
Cd2—O7	2.2795 (11)
Cd2—O9	2.2675 (11)
Cd2—O10	2.6839 (12)
Cd2—O13	2.3486 (12)
Cd2—O14	2.2953 (12)
Cd2—N3	2.2824 (13)

**Table 2:** Hydrogen-bond geometry (Å, °) *Cg*1 is the centroid of the C2–C7 ring.

*D*—H⋯*A*	*D*—H	H⋯*A*	*D*⋯*A*	*D*—H⋯*A*
N2—H2*A*⋯O11^i^	0.88	2.21	3.025 (2)	154
N2—H2*B*⋯O1^ii^	0.88	2.23	3.054 (2)	156
N4—H4*B*⋯O13^iii^	0.88	2.13	2.937 (2)	151
O3—H3⋯O2	0.84	1.81	2.548 (2)	146
O5—H51⋯O7^iv^	0.78 (3)	1.95 (3)	2.722 (2)	172 (3)
O5—H52⋯O1^v^	0.82 (3)	1.89 (3)	2.687 (2)	165 (3)
O8—H81⋯O6	0.84	1.83	2.569 (2)	146
O11—H111⋯O5^vi^	0.84	2.52	3.048 (2)	122
O11—H111⋯O9	0.84	1.79	2.535 (2)	146
O13—H131⋯O3^vi^	0.76 (3)	2.02 (3)	2.760 (2)	165 (2)
O13—H132⋯O4^vii^	0.79 (3)	1.88 (3)	2.656 (2)	168 (3)
O14—H141⋯O15^ii^	0.78 (3)	1.92 (3)	2.693 (2)	178.1 (5)
O14—H142⋯O10^viii^	0.84 (3)	1.89 (3)	2.720 (2)	178 (4)
O15—H15*A*⋯O16*A*	0.86 (2)	1.95 (2)	2.764 (4)	156 (2)
O15—H15*A*⋯O16*B*	0.86 (2)	1.93 (2)	2.689 (5)	146 (2)
O15—H15*B*⋯O12	0.84 (3)	2.08 (3)	2.880 (2)	159 (3)
O16*A*—H161⋯O8^vii^	0.83 (5)	2.53 (5)	3.139 (4)	132 (4)
O16*A*—H162⋯O1^ix^	0.89 (4)	2.14 (3)	2.965 (4)	153 (5)
O16*B*—H164⋯O8	0.91 (2)	1.91 (2)	2.748 (2)	153 (3)
C28—H28⋯O6	0.95	2.35	3.101 (2)	136
N4—H4*A*⋯*Cg*1	0.88	2.69	3.470 (2)	148

Symmetry codes: (i) 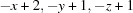; (ii) 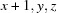; (iii) 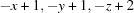; (iv) 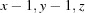; (v) 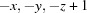; (vi) 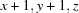; (vii) 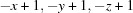; (viii) 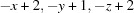; (ix) 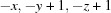.

**Fig. 1.**
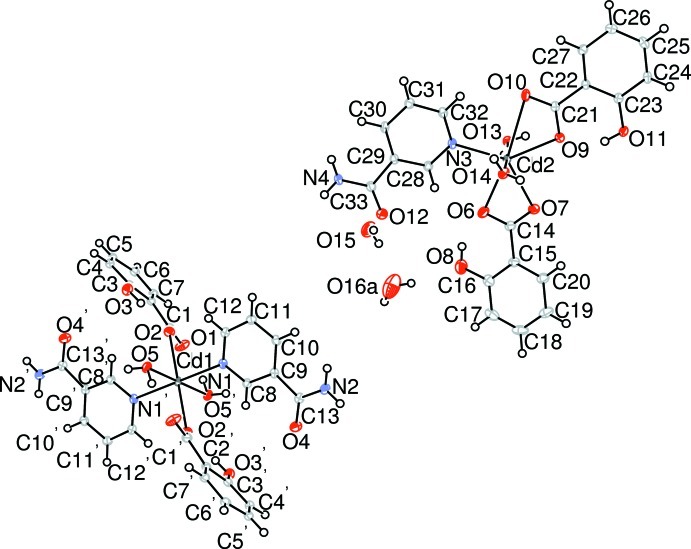
The molecular structure of the title molecule with the atom-numbering scheme. Displacement ellipsoids are drawn at the 50% probability level.Only one of the disordered water molecules is shown for clarity. Primed atoms are generated by the symmetry operator: (') - x, - y, - z.

## supplementary crystallographic information

### Comment

As part of our ongoing study on transition metal complexes of benzoate and nicotinamide, (NA), herein we report the synthesis and the structure of the title cocrystal diaquabis(salicylato-κ*O*)bis(nicotinamide-κ*N*) cadmium(II),(A), and diaquabis(salicylato-κ^2^*O*;*O*') (nicotinamide-κ*N*)cadmium(II)dihydrate, (B).

The components of the title compound, [Cd(C_7_H_5_O_3_)_2_(C_6_H_6_NO)_2_ (H_2_O)_2_], (A), and [Cd(C_7_H_5_O_3_)_2_(C_6_H_6_NO)(H_2_O)_2_].2(H_2_O), (B), are mononuclear complexes. In complex A, the Cd^II^ cation is located on an inversion center and is coordinated by two salicylate anions, two nicotinamide (NA) ligands and two water molecules in a slightly distorted octahedral geometry (Fig. 1). In complex B, the Cd^II^ cation is coordinated by two salicylate anions, one nicotinamide (NA) ligand and two water molecules completing the irregular seven-coordination geometry (Fig. 1). There are extensive intramolecular O—H···O and weak C—H···O hydrogen bonding, beside of the extensive intermolecular O—H···O and N—H···O hydrogen bonding (Table 2) in the crystal structure.

The average Cd—O bond lengths (Table 1) are 2.3240 (12) and 2.4094 (12) Å for (A) and (B), respectively, and the Cd atoms are displaced out of the least-squares planes of the carboxylate groups: Cd1 atom for (O1/C1/O2) by 0.7250 (1) Å, Cd2 atom for (O6/C14/O7) and (O9/C21/O10) by -0.3415 (1) and -0.1105 (1) Å, respectively. In (B), the O6—Cd2—O7 and O9—Cd2—O10 angles are 53.45 (4) and 51.97 (4) °, respectively. The corresponding O—M—O (where *M* is a metal) angles are 52.91 (4)° and 53.96 (4)° in [Cd(C_8_H_5_O_3_)_2_(C_6_H_6_N_2_O)_2_(H_2_O)].H_2_O (Hökelek *et al.*, 2009*a*), 60.70 (4)° in [Co(C_9_H_10_NO_2_)_2_(C_6_H_6_N_2_O)(H_2_O)_2_] (Hökelek *et al.*, 2009*b*), 58.45 (9)° in [Mn(C_9_H_10_NO_2_)_2_- (C_6_H_6_N_2_O)(H_2_O)_2_] (Hökelek *et al.*, 2009*c*), 60.03 (6)° in [Zn(C_8_H_8_NO_2_)_2_(C_6_H_6_N_2_O)_2_].H_2_O (Hökelek *et al.*, 2009*d*), 58.3 (3)° in [Zn_2_(DENA)_2_(C_7_H_5_O_3_)_4_].2H_2_O (Hökelek & Necefoğlu, 1996) and 55.2 (1)° in [Cu(Asp)_2_(py)_2_] (where Asp is acetylsalicylate and py is pyridine) (Greenaway *et al.*, 1984).

The dihedral angles between the planar carboxylate groups and the adjacent benzene rings A (C2—C7), C (C15—C20) and D (C22—C27) are 16.26 (17), 5.32 (16) and 3.53 (12) °, respectively, while those between rings A, B (N1/C8—C12) and C, D, E (N3/C28—C32), F (Cd2/O6/O7/C14), G (Cd2/O9/O10/C21) are A/B = 73.75 (4), C/D = 24.80 (6), C/E = 30.95 (6), D/E = 6.88 (6) and F/G = 25.62 (5) °.

In the crystal structure, extensive O—H···O and N—H···O hydrogen bonding (Table 2) may be effective in the stabilization of the structure. π···π contacts between the pyridine and benzene rings Cg2—Cg3^i^, between the benzene rings Cg3—Cg3^i^, between the benzene and pyridine rings Cg4—Cg5^ii^ and between the pyridine rings Cg5—Cg5^iii^, [symmetry codes: (i) -x, 1 - y, 1 - z, (ii) -x, 1 - y, -z, (iii) 1 - x, 1 - y, -z, where Cg2, Cg3, Cg4 and Cg5 are the centroids of the rings B (N1/C8—C12), C (C15—C20), D (C22—C27) and E (N3/C28—C32), respectively] may further stabilize the structure, with centroid-centroid distances of 3.5989 (10), 3.6005 (10), 3.5800 (9) and 3.5205 (10) Å, respectively]. A weak C-H···π interaction also occurs in the crsytal.

### Experimental

The title compound was prepared by the reaction of 3CdSO_4_.8H_2_O (1.283 g, 5 mmol) in H_2_O (50 ml) and NA (1.220 g, 10 mmol) in H_2_O (20 ml) with sodium salicylate (1.601 g, 10 mmol) in H_2_O (200 ml). The mixture was filtered and set aside to crystallize at ambient temperature for two weeks, giving colorless single crystals.

### Refinement

Water H atoms were located in a difference Fourier map and refined isotropically. The C, N and O -bound H-atoms were positioned geometrically with C—H = 0.95, N—H = 0.88 and O—H = 0.84 Å for aromatic, NH_2_ and OH H atoms, respectively, and constrained to ride on their parent atoms, with *U*_iso_(H) = k × *U*_eq_(C,N,O), where k = 1.5 for OH H-atoms and k = 1.2 for all other H-atoms. During the refinement process the disordered O16A, H161, H162 and O16B, H163, H164 atoms were refined with occupancies ratios of 0.70:0.30.

### Crystal data

**Table:**

[Cd(C_7_H_5_O_3_)_2_(C_6_H_6_NO)_2_(H_2_O)_2_]·2[Cd(C_7_H_5_O_3_)_2_(C_6_H_6_NO) (H_2_O)_2_]·4H_2_O	*Z* = 1
*M**_r_* = 1828.56	*F*(000) = 926
Triclinic, *P*1	*D*_x_ = 1.713 Mg m^−^^3^
Hall symbol: -P 1	Mo *K*α radiation, λ = 0.71073 Å
*a* = 10.3446 (2) Å	Cell parameters from 9969 reflections
*b* = 13.5779 (3) Å	θ = 2.3–28.4°
*c* = 14.6586 (3) Å	µ = 0.99 mm^−^^1^
α = 71.226 (3)°	*T* = 100 K
β = 71.364 (3)°	Block, colorless
γ = 69.221 (2)°	0.42 × 0.32 × 0.29 mm
*V* = 1772.85 (7) Å^3^	

### Data collection

**Table:**

Bruker Kappa APEXII CCD area-detector diffractometer	8814 independent reflections
Radiation source: fine-focus sealed tube	8335 reflections with *I* > 2σ(*I*)
Graphite monochromator	*R*_int_ = 0.019
φ and ω scans	θ_max_ = 28.4°, θ_min_ = 1.5°
Absorption correction: multi-scan (*SADABS*; Bruker, 2005)	*h* = −13→13
*T*_min_ = 0.691, *T*_max_ = 0.751	*k* = −17→18
31862 measured reflections	*l* = −19→19

### Refinement

**Table:**

Refinement on *F*^2^	Primary atom site location: structure-invariant direct methods
Least-squares matrix: full	Secondary atom site location: difference Fourier map
*R*[*F*^2^ > 2σ(*F*^2^)] = 0.019	Hydrogen site location: inferred from neighbouring sites
*wR*(*F*^2^) = 0.050	H atoms treated by a mixture of independent and constrained refinement
*S* = 1.07	*w* = 1/[σ^2^(*F*_o_^2^) + (0.0186*P*)^2^ + 1.5957*P*] where *P* = (*F*_o_^2^ + 2*F*_c_^2^)/3
8814 reflections	(Δ/σ)_max_ = 0.020
545 parameters	Δρ_max_ = 0.78 e Å^−^^3^
12 restraints	Δρ_min_ = −0.55 e Å^−^^3^

### Special details

**Table:**

Geometry. All esds (except the esd in the dihedral angle between two l.s. planes) are estimated using the full covariance matrix. The cell esds are taken into account individually in the estimation of esds in distances, angles and torsion angles; correlations between esds in cell parameters are only used when they are defined by crystal symmetry. An approximate (isotropic) treatment of cell esds is used for estimating esds involving l.s. planes.
Refinement. Refinement of F^2^ against ALL reflections. The weighted R-factor wR and goodness of fit S are based on F^2^, conventional R-factors R are based on F, with F set to zero for negative F^2^. The threshold expression of F^2^ > 2sigma(F^2^) is used only for calculating R-factors(gt) etc. and is not relevant to the choice of reflections for refinement. R-factors based on F^2^ are statistically about twice as large as those based on F, and R- factors based on ALL data will be even larger.

### Fractional atomic coordinates and isotropic or equivalent isotropic displacement parameters (Å^2^)

**Table:**

	*x*	*y*	*z*	*U*_iso_*/*U*_eq_	Occ. (<1)
Cd1	0.0000	0.0000	0.5000	0.01311 (4)	
Cd2	0.877004 (11)	0.624380 (8)	0.830132 (7)	0.01203 (3)	
O1	−0.31150 (13)	0.19557 (12)	0.54455 (9)	0.0269 (3)	
O2	−0.16718 (12)	0.06304 (10)	0.63352 (8)	0.0180 (2)	
O3	−0.27174 (12)	−0.03188 (9)	0.80840 (9)	0.0194 (2)	
H3	−0.2076	−0.0189	0.7578	0.029*	
O4	0.49310 (12)	0.13733 (10)	0.28241 (8)	0.0201 (2)	
O5	0.08538 (14)	−0.16188 (10)	0.60766 (9)	0.0178 (2)	
H51	0.044 (3)	−0.205 (2)	0.6227 (19)	0.039 (7)*	
H52	0.156 (3)	−0.184 (2)	0.566 (2)	0.038 (7)*	
O6	0.77699 (14)	0.61782 (10)	0.69250 (9)	0.0224 (2)	
O7	0.96639 (14)	0.67201 (10)	0.66363 (8)	0.0211 (2)	
O8	0.70522 (15)	0.59461 (12)	0.54991 (11)	0.0310 (3)	
H81	0.6944	0.6014	0.6071	0.046*	
O9	0.98282 (12)	0.74204 (9)	0.83579 (8)	0.0164 (2)	
O10	0.90014 (12)	0.66520 (9)	0.99076 (8)	0.0182 (2)	
O11	1.13543 (13)	0.86917 (10)	0.78926 (8)	0.0189 (2)	
H111	1.0909	0.8337	0.7797	0.028*	
O12	0.58701 (14)	0.36892 (11)	0.80202 (9)	0.0258 (3)	
O13	0.67334 (13)	0.77253 (10)	0.84526 (9)	0.0162 (2)	
H131	0.700 (2)	0.820 (2)	0.8404 (17)	0.025 (6)*	
H132	0.630 (3)	0.792 (2)	0.8042 (19)	0.031 (6)*	
O14	1.07045 (13)	0.47416 (10)	0.83130 (9)	0.0174 (2)	
H141	1.146 (3)	0.480 (2)	0.803 (2)	0.038 (7)*	
H142	1.078 (3)	0.431 (2)	0.886 (2)	0.035 (6)*	
O15	0.33286 (16)	0.49084 (15)	0.73091 (12)	0.0383 (4)	
H15A	0.331 (3)	0.532 (2)	0.6724 (14)	0.056 (9)*	
H15B	0.416 (2)	0.454 (2)	0.736 (2)	0.055*	
O16A	0.3642 (4)	0.5663 (3)	0.5283 (3)	0.0691 (10)	0.70
H161	0.330 (5)	0.563 (4)	0.486 (3)	0.070*	0.70
H162	0.377 (5)	0.632 (2)	0.510 (3)	0.070*	0.70
O16B	0.4434 (6)	0.5903 (5)	0.5461 (4)	0.0407 (12)	0.30
H163	0.478 (9)	0.541 (6)	0.518 (7)	0.055*	0.30
H164	0.516 (6)	0.615 (7)	0.543 (7)	0.055*	0.30
N1	0.16652 (14)	0.06569 (10)	0.51738 (10)	0.0141 (2)	
N2	0.59990 (15)	0.17238 (12)	0.37443 (10)	0.0201 (3)	
H2A	0.6712	0.1831	0.3237	0.024*	
H2B	0.5985	0.1786	0.4327	0.024*	
N3	0.75275 (14)	0.50506 (10)	0.93253 (10)	0.0137 (2)	
N4	0.47937 (16)	0.27600 (12)	0.94629 (11)	0.0205 (3)	
H4A	0.4403	0.2514	0.9162	0.025*	
H4B	0.4640	0.2578	1.0113	0.025*	
C1	−0.28699 (17)	0.13245 (14)	0.62445 (12)	0.0171 (3)	
C2	−0.40629 (16)	0.13224 (13)	0.71493 (11)	0.0146 (3)	
C3	−0.39584 (16)	0.04683 (13)	0.79939 (11)	0.0147 (3)	
C4	−0.51546 (17)	0.03905 (13)	0.87677 (11)	0.0165 (3)	
H4	−0.5096	−0.0216	0.9319	0.020*	
C5	−0.64234 (17)	0.11948 (13)	0.87315 (12)	0.0171 (3)	
H5	−0.7235	0.1140	0.9260	0.021*	
C6	−0.65202 (17)	0.20861 (13)	0.79242 (12)	0.0166 (3)	
H6	−0.7381	0.2655	0.7914	0.020*	
C7	−0.53499 (17)	0.21351 (13)	0.71370 (11)	0.0159 (3)	
H7	−0.5424	0.2732	0.6578	0.019*	
C8	0.27348 (16)	0.09059 (12)	0.44208 (11)	0.0141 (3)	
H8	0.2780	0.0842	0.3784	0.017*	
C9	0.37801 (16)	0.12524 (12)	0.45284 (11)	0.0133 (3)	
C10	0.37015 (17)	0.13455 (12)	0.54640 (11)	0.0152 (3)	
H10	0.4399	0.1577	0.5567	0.018*	
C11	0.25952 (17)	0.10974 (13)	0.62432 (11)	0.0166 (3)	
H11	0.2521	0.1159	0.6887	0.020*	
C12	0.15991 (17)	0.07589 (12)	0.60704 (11)	0.0156 (3)	
H12	0.0840	0.0592	0.6606	0.019*	
C13	0.49426 (16)	0.14670 (12)	0.36310 (11)	0.0149 (3)	
C14	0.88452 (18)	0.64560 (12)	0.63308 (11)	0.0174 (3)	
C15	0.91699 (18)	0.64417 (12)	0.52719 (11)	0.0171 (3)	
C16	0.82761 (19)	0.61560 (13)	0.49183 (13)	0.0205 (3)	
C17	0.8645 (2)	0.60858 (14)	0.39349 (13)	0.0255 (4)	
H17	0.8043	0.5891	0.3693	0.031*	
C18	0.9885 (2)	0.62997 (14)	0.33131 (13)	0.0274 (4)	
H18	1.0134	0.6242	0.2646	0.033*	
C19	1.0772 (2)	0.65980 (15)	0.36474 (13)	0.0255 (4)	
H19	1.1616	0.6755	0.3211	0.031*	
C20	1.04131 (19)	0.66644 (14)	0.46248 (12)	0.0207 (3)	
H20	1.1019	0.6864	0.4859	0.025*	
C21	0.96738 (16)	0.72866 (12)	0.92860 (11)	0.0130 (3)	
C22	1.03321 (15)	0.79060 (12)	0.95921 (11)	0.0124 (3)	
C23	1.11546 (16)	0.85634 (12)	0.88791 (11)	0.0140 (3)	
C24	1.18204 (17)	0.90993 (13)	0.91789 (12)	0.0177 (3)	
H24	1.2383	0.9537	0.8697	0.021*	
C25	1.16626 (18)	0.89943 (13)	1.01733 (12)	0.0187 (3)	
H25	1.2112	0.9366	1.0372	0.022*	
C26	1.08496 (18)	0.83482 (13)	1.08907 (12)	0.0183 (3)	
H26	1.0741	0.8280	1.1575	0.022*	
C27	1.02046 (16)	0.78079 (13)	1.05937 (11)	0.0150 (3)	
H27	0.9662	0.7360	1.1082	0.018*	
C28	0.69587 (16)	0.45880 (12)	0.89278 (11)	0.0147 (3)	
H28	0.7059	0.4791	0.8231	0.018*	
C29	0.62276 (16)	0.38230 (12)	0.94916 (11)	0.0136 (3)	
C30	0.60890 (16)	0.35230 (12)	1.05111 (11)	0.0146 (3)	
H30	0.5601	0.3000	1.0917	0.018*	
C31	0.66750 (17)	0.39997 (13)	1.09260 (11)	0.0156 (3)	
H31	0.6592	0.3810	1.1621	0.019*	
C32	0.73819 (16)	0.47544 (12)	1.03128 (11)	0.0148 (3)	
H32	0.7782	0.5078	1.0601	0.018*	
C33	0.56221 (17)	0.34110 (13)	0.89312 (12)	0.0173 (3)	

### Atomic displacement parameters (Å^2^)

**Table:**

	*U*^11^	*U*^22^	*U*^33^	*U*^12^	*U*^13^	*U*^23^
Cd1	0.01042 (7)	0.01605 (8)	0.01411 (7)	−0.00624 (6)	−0.00107 (5)	−0.00409 (5)
Cd2	0.01288 (6)	0.01225 (5)	0.01173 (5)	−0.00601 (4)	−0.00242 (4)	−0.00147 (4)
O1	0.0173 (6)	0.0418 (8)	0.0148 (5)	−0.0056 (6)	−0.0013 (5)	−0.0033 (5)
O2	0.0125 (5)	0.0234 (6)	0.0191 (5)	−0.0054 (5)	−0.0004 (4)	−0.0093 (5)
O3	0.0150 (6)	0.0182 (6)	0.0216 (6)	−0.0033 (5)	−0.0025 (4)	−0.0037 (4)
O4	0.0148 (6)	0.0300 (6)	0.0164 (5)	−0.0068 (5)	−0.0036 (4)	−0.0060 (5)
O5	0.0169 (6)	0.0215 (6)	0.0154 (5)	−0.0100 (5)	−0.0015 (4)	−0.0019 (4)
O6	0.0267 (7)	0.0206 (6)	0.0168 (5)	−0.0099 (5)	0.0013 (5)	−0.0029 (4)
O7	0.0314 (7)	0.0205 (6)	0.0131 (5)	−0.0117 (5)	−0.0055 (5)	−0.0012 (4)
O8	0.0296 (7)	0.0337 (7)	0.0319 (7)	−0.0148 (6)	−0.0089 (6)	−0.0023 (6)
O9	0.0177 (6)	0.0203 (6)	0.0145 (5)	−0.0094 (5)	−0.0030 (4)	−0.0046 (4)
O10	0.0191 (6)	0.0188 (6)	0.0190 (5)	−0.0118 (5)	−0.0062 (4)	0.0017 (4)
O11	0.0224 (6)	0.0246 (6)	0.0129 (5)	−0.0149 (5)	−0.0023 (4)	−0.0010 (4)
O12	0.0301 (7)	0.0368 (7)	0.0179 (6)	−0.0202 (6)	−0.0014 (5)	−0.0079 (5)
O13	0.0166 (6)	0.0159 (6)	0.0172 (5)	−0.0053 (5)	−0.0064 (4)	−0.0020 (4)
O14	0.0162 (6)	0.0175 (6)	0.0157 (5)	−0.0047 (5)	−0.0033 (4)	−0.0006 (4)
O15	0.0264 (8)	0.0539 (10)	0.0326 (8)	−0.0164 (7)	−0.0057 (6)	−0.0029 (7)
O16A	0.0534 (18)	0.069 (2)	0.066 (2)	−0.0221 (16)	−0.0284 (15)	0.0282 (16)
O16B	0.040 (3)	0.043 (3)	0.036 (3)	−0.012 (3)	−0.016 (2)	0.004 (2)
N1	0.0136 (6)	0.0134 (6)	0.0158 (6)	−0.0050 (5)	−0.0040 (5)	−0.0021 (5)
N2	0.0150 (7)	0.0312 (8)	0.0176 (6)	−0.0129 (6)	−0.0009 (5)	−0.0061 (6)
N3	0.0114 (6)	0.0122 (6)	0.0176 (6)	−0.0039 (5)	−0.0031 (5)	−0.0033 (5)
N4	0.0248 (7)	0.0245 (7)	0.0193 (6)	−0.0165 (6)	−0.0025 (6)	−0.0064 (5)
C1	0.0149 (7)	0.0233 (8)	0.0161 (7)	−0.0082 (6)	−0.0010 (6)	−0.0080 (6)
C2	0.0136 (7)	0.0183 (7)	0.0148 (7)	−0.0069 (6)	−0.0014 (5)	−0.0070 (6)
C3	0.0140 (7)	0.0153 (7)	0.0177 (7)	−0.0045 (6)	−0.0035 (6)	−0.0075 (6)
C4	0.0188 (8)	0.0166 (7)	0.0156 (7)	−0.0079 (6)	−0.0023 (6)	−0.0042 (6)
C5	0.0164 (7)	0.0214 (8)	0.0167 (7)	−0.0096 (6)	0.0017 (6)	−0.0093 (6)
C6	0.0142 (7)	0.0192 (8)	0.0187 (7)	−0.0046 (6)	−0.0029 (6)	−0.0086 (6)
C7	0.0165 (7)	0.0182 (7)	0.0155 (7)	−0.0063 (6)	−0.0037 (6)	−0.0055 (6)
C8	0.0135 (7)	0.0152 (7)	0.0144 (7)	−0.0048 (6)	−0.0043 (5)	−0.0026 (5)
C9	0.0110 (7)	0.0120 (7)	0.0161 (7)	−0.0026 (5)	−0.0043 (5)	−0.0019 (5)
C10	0.0146 (7)	0.0155 (7)	0.0174 (7)	−0.0049 (6)	−0.0063 (6)	−0.0029 (6)
C11	0.0187 (8)	0.0178 (7)	0.0140 (7)	−0.0057 (6)	−0.0052 (6)	−0.0027 (6)
C12	0.0155 (7)	0.0148 (7)	0.0154 (7)	−0.0055 (6)	−0.0027 (6)	−0.0018 (5)
C13	0.0122 (7)	0.0138 (7)	0.0165 (7)	−0.0021 (6)	−0.0040 (5)	−0.0018 (5)
C14	0.0246 (8)	0.0112 (7)	0.0135 (7)	−0.0039 (6)	−0.0032 (6)	−0.0015 (5)
C15	0.0234 (8)	0.0123 (7)	0.0130 (7)	−0.0029 (6)	−0.0048 (6)	−0.0015 (5)
C16	0.0250 (9)	0.0133 (7)	0.0225 (8)	−0.0030 (6)	−0.0099 (7)	−0.0013 (6)
C17	0.0386 (11)	0.0167 (8)	0.0250 (8)	−0.0024 (7)	−0.0191 (8)	−0.0041 (6)
C18	0.0436 (11)	0.0180 (8)	0.0150 (7)	0.0017 (8)	−0.0111 (7)	−0.0040 (6)
C19	0.0307 (10)	0.0221 (8)	0.0149 (7)	−0.0034 (7)	−0.0009 (7)	−0.0017 (6)
C20	0.0253 (9)	0.0185 (8)	0.0156 (7)	−0.0048 (7)	−0.0042 (6)	−0.0024 (6)
C21	0.0100 (7)	0.0119 (7)	0.0163 (7)	−0.0020 (5)	−0.0033 (5)	−0.0030 (5)
C22	0.0097 (7)	0.0120 (6)	0.0151 (7)	−0.0028 (5)	−0.0029 (5)	−0.0032 (5)
C23	0.0130 (7)	0.0130 (7)	0.0152 (7)	−0.0036 (6)	−0.0032 (5)	−0.0026 (5)
C24	0.0176 (8)	0.0149 (7)	0.0219 (8)	−0.0085 (6)	−0.0047 (6)	−0.0017 (6)
C25	0.0200 (8)	0.0160 (7)	0.0246 (8)	−0.0060 (6)	−0.0084 (6)	−0.0064 (6)
C26	0.0197 (8)	0.0191 (8)	0.0166 (7)	−0.0033 (6)	−0.0054 (6)	−0.0063 (6)
C27	0.0130 (7)	0.0151 (7)	0.0152 (7)	−0.0037 (6)	−0.0018 (5)	−0.0030 (5)
C28	0.0135 (7)	0.0150 (7)	0.0155 (7)	−0.0047 (6)	−0.0016 (5)	−0.0042 (5)
C29	0.0100 (7)	0.0129 (7)	0.0179 (7)	−0.0032 (5)	−0.0014 (5)	−0.0055 (5)
C30	0.0116 (7)	0.0129 (7)	0.0181 (7)	−0.0043 (6)	−0.0020 (5)	−0.0027 (5)
C31	0.0146 (7)	0.0157 (7)	0.0159 (7)	−0.0044 (6)	−0.0036 (6)	−0.0026 (6)
C32	0.0130 (7)	0.0147 (7)	0.0180 (7)	−0.0041 (6)	−0.0049 (6)	−0.0039 (6)
C33	0.0159 (7)	0.0191 (8)	0.0190 (7)	−0.0069 (6)	−0.0016 (6)	−0.0075 (6)

### Geometric parameters (Å, º)

**Table:**

Cd1—O2	2.3279 (11)	C1—C2	1.495 (2)
Cd1—O2^i^	2.3279 (11)	C2—C7	1.397 (2)
Cd1—O5	2.3200 (12)	C2—C3	1.401 (2)
Cd1—O5^i^	2.3200 (12)	C3—C4	1.396 (2)
Cd1—N1	2.3118 (13)	C4—C5	1.381 (2)
Cd1—N1^i^	2.3118 (13)	C4—H4	0.9500
Cd2—O6	2.5814 (13)	C5—C6	1.394 (2)
Cd2—O7	2.2795 (11)	C5—H5	0.9500
Cd2—O9	2.2675 (11)	C6—C7	1.384 (2)
Cd2—O10	2.6839 (12)	C6—H6	0.9500
Cd2—O13	2.3486 (12)	C7—H7	0.9500
Cd2—O14	2.2953 (12)	C8—H8	0.9500
Cd2—N3	2.2824 (13)	C9—C8	1.390 (2)
O1—C1	1.249 (2)	C9—C13	1.501 (2)
O2—C1	1.280 (2)	C10—C9	1.392 (2)
O3—C3	1.3608 (19)	C10—H10	0.9500
O3—H3	0.8400	C11—C10	1.385 (2)
O4—C13	1.2338 (19)	C11—H11	0.9500
O5—H51	0.78 (3)	C12—C11	1.384 (2)
O5—H52	0.83 (3)	C12—H12	0.9500
O6—C14	1.267 (2)	C14—C15	1.485 (2)
O7—C14	1.268 (2)	C15—C20	1.399 (2)
O8—C16	1.349 (2)	C15—C16	1.401 (2)
O8—H81	0.8400	C16—C17	1.394 (2)
O9—C21	1.2774 (18)	C17—C18	1.381 (3)
O10—C21	1.2535 (18)	C17—H17	0.9500
O11—C23	1.3552 (18)	C18—C19	1.388 (3)
O11—H111	0.8400	C18—H18	0.9500
O12—C33	1.232 (2)	C19—C20	1.385 (2)
O13—H131	0.76 (2)	C19—H19	0.9500
O13—H132	0.79 (3)	C20—H20	0.9500
O14—H141	0.78 (3)	C21—C22	1.489 (2)
O14—H142	0.83 (3)	C22—C27	1.398 (2)
O15—H15A	0.863 (16)	C22—C23	1.408 (2)
O15—H15B	0.843 (17)	C23—C24	1.397 (2)
O16A—O16B	1.108 (7)	C24—C25	1.379 (2)
O16A—H161	0.829 (19)	C24—H24	0.9500
O16A—H162	0.895 (17)	C25—C26	1.396 (2)
O16A—H163	1.08 (8)	C25—H25	0.9500
O16B—H162	0.92 (5)	C26—C27	1.382 (2)
O16B—H163	0.81 (2)	C26—H26	0.9500
O16B—H164	0.909 (19)	C27—H27	0.9500
N1—C8	1.342 (2)	C28—C29	1.393 (2)
N1—C12	1.343 (2)	C28—H28	0.9500
N2—C13	1.329 (2)	C29—C30	1.390 (2)
N2—H2A	0.8800	C29—C33	1.501 (2)
N2—H2B	0.8800	C30—C31	1.387 (2)
N3—C28	1.3405 (19)	C30—H30	0.9500
N3—C32	1.345 (2)	C31—C32	1.383 (2)
N4—C33	1.338 (2)	C31—H31	0.9500
N4—H4A	0.8800	C32—H32	0.9500
N4—H4B	0.8800		
			
O2—Cd1—O2^i^	180.0	C6—C7—C2	121.19 (15)
O5—Cd1—O2	89.71 (4)	C6—C7—H7	119.4
O5^i^—Cd1—O2	90.29 (4)	N1—C8—C9	123.14 (14)
O5—Cd1—O2^i^	90.29 (4)	N1—C8—H8	118.4
O5^i^—Cd1—O2^i^	89.71 (4)	C9—C8—H8	118.4
O5—Cd1—O5^i^	180.00 (6)	C8—C9—C10	117.94 (14)
N1—Cd1—O2	91.83 (4)	C8—C9—C13	117.52 (13)
N1^i^—Cd1—O2	88.17 (4)	C10—C9—C13	124.51 (14)
N1—Cd1—O2^i^	88.17 (4)	C9—C10—H10	120.4
N1^i^—Cd1—O2^i^	91.83 (4)	C11—C10—C9	119.25 (14)
N1—Cd1—O5	88.80 (4)	C11—C10—H10	120.4
N1^i^—Cd1—O5	91.20 (4)	C10—C11—H11	120.5
N1—Cd1—O5^i^	91.20 (4)	C12—C11—C10	119.01 (14)
N1^i^—Cd1—O5^i^	88.80 (4)	C12—C11—H11	120.5
N1—Cd1—N1^i^	180.00 (5)	N1—C12—C11	122.49 (14)
O7—Cd2—O6	53.45 (4)	N1—C12—H12	118.8
O7—Cd2—O13	99.00 (4)	C11—C12—H12	118.8
O7—Cd2—O14	86.39 (5)	O4—C13—N2	121.54 (15)
O7—Cd2—N3	135.25 (4)	O4—C13—C9	120.59 (14)
O9—Cd2—O6	130.94 (4)	N2—C13—C9	117.83 (14)
O9—Cd2—O7	83.88 (4)	O6—C14—O7	120.68 (14)
O9—Cd2—O10	51.97 (4)	O6—C14—C15	119.54 (15)
O9—Cd2—O13	82.42 (4)	O7—C14—C15	119.77 (15)
O9—Cd2—O14	97.85 (4)	C16—C15—C14	120.68 (15)
O9—Cd2—N3	140.77 (4)	C20—C15—C14	120.08 (15)
O13—Cd2—O6	81.90 (4)	C20—C15—C16	119.17 (15)
O14—Cd2—O6	101.78 (4)	O8—C16—C15	122.18 (16)
O14—Cd2—O13	174.60 (4)	O8—C16—C17	118.09 (16)
N3—Cd2—O6	85.89 (4)	C17—C16—C15	119.72 (17)
N3—Cd2—O13	91.82 (4)	C16—C17—H17	120.0
N3—Cd2—O14	84.56 (5)	C18—C17—C16	120.01 (17)
C1—O2—Cd1	122.27 (10)	C18—C17—H17	120.0
C3—O3—H3	109.5	C17—C18—C19	121.02 (16)
Cd1—O5—H51	116.4 (19)	C17—C18—H18	119.5
Cd1—O5—H52	94.7 (18)	C19—C18—H18	119.5
H52—O5—H51	103 (3)	C18—C19—H19	120.4
C14—O6—Cd2	85.62 (10)	C20—C19—C18	119.16 (17)
C14—O7—Cd2	99.61 (10)	C20—C19—H19	120.4
C16—O8—H81	109.5	C15—C20—H20	119.5
C21—O9—Cd2	103.02 (9)	C19—C20—C15	120.90 (17)
C23—O11—H111	109.5	C19—C20—H20	119.5
Cd2—O13—H131	105.6 (18)	O9—C21—C22	117.30 (13)
Cd2—O13—H132	116.8 (18)	O10—C21—O9	120.87 (14)
H131—O13—H132	105 (2)	O10—C21—C22	121.83 (13)
Cd2—O14—H141	119.8 (19)	C23—C22—C21	120.72 (13)
Cd2—O14—H142	117.3 (17)	C27—C22—C21	120.76 (13)
H142—O14—H141	108 (2)	C27—C22—C23	118.45 (14)
H15A—O15—H15B	112 (3)	O11—C23—C22	122.46 (13)
H162—O16A—H161	106 (3)	O11—C23—C24	117.49 (14)
H163—O16B—H164	106 (5)	C24—C23—C22	120.04 (14)
C8—N1—Cd1	122.70 (10)	C23—C24—H24	119.9
C8—N1—C12	118.15 (13)	C25—C24—C23	120.11 (14)
C12—N1—Cd1	119.10 (10)	C25—C24—H24	119.9
C13—N2—H2A	120.0	C24—C25—C26	120.68 (15)
C13—N2—H2B	120.0	C24—C25—H25	119.7
H2A—N2—H2B	120.0	C26—C25—H25	119.7
C28—N3—Cd2	118.96 (10)	C25—C26—H26	120.4
C28—N3—C32	117.99 (13)	C27—C26—C25	119.19 (14)
C32—N3—Cd2	123.00 (10)	C27—C26—H26	120.4
C33—N4—H4A	120.0	C22—C27—H27	119.2
C33—N4—H4B	120.0	C26—C27—C22	121.52 (14)
H4A—N4—H4B	120.0	C26—C27—H27	119.2
O1—C1—O2	124.22 (15)	N3—C28—C29	122.91 (14)
O1—C1—C2	118.68 (15)	N3—C28—H28	118.5
O2—C1—C2	117.00 (14)	C29—C28—H28	118.5
C3—C2—C1	121.07 (14)	C28—C29—C33	115.98 (14)
C7—C2—C1	120.12 (14)	C30—C29—C28	118.44 (14)
C7—C2—C3	118.61 (14)	C30—C29—C33	125.56 (14)
O3—C3—C2	122.00 (14)	C29—C30—H30	120.6
O3—C3—C4	117.82 (14)	C31—C30—C29	118.87 (14)
C4—C3—C2	120.17 (15)	C31—C30—H30	120.6
C3—C4—H4	120.0	C30—C31—H31	120.5
C5—C4—C3	120.01 (15)	C32—C31—C30	118.99 (14)
C5—C4—H4	120.0	C32—C31—H31	120.5
C4—C5—C6	120.42 (15)	N3—C32—C31	122.80 (14)
C4—C5—H5	119.8	N3—C32—H32	118.6
C6—C5—H5	119.8	C31—C32—H32	118.6
C5—C6—H6	120.3	O12—C33—N4	122.50 (15)
C7—C6—C5	119.38 (15)	O12—C33—C29	120.23 (14)
C7—C6—H6	120.3	N4—C33—C29	117.26 (14)
C2—C7—H7	119.4		
			
O5—Cd1—O2—C1	−151.77 (12)	C7—C2—C3—O3	−176.12 (13)
O5^i^—Cd1—O2—C1	28.23 (12)	C7—C2—C3—C4	5.3 (2)
N1—Cd1—O2—C1	119.44 (12)	C1—C2—C7—C6	172.72 (14)
N1^i^—Cd1—O2—C1	−60.56 (12)	C3—C2—C7—C6	−2.3 (2)
O2—Cd1—N1—C8	−155.47 (12)	O3—C3—C4—C5	177.09 (14)
O2^i^—Cd1—N1—C8	24.53 (12)	C2—C3—C4—C5	−4.3 (2)
O2—Cd1—N1—C12	27.03 (12)	C3—C4—C5—C6	0.1 (2)
O2^i^—Cd1—N1—C12	−152.97 (12)	C4—C5—C6—C7	2.9 (2)
O5—Cd1—N1—C8	114.85 (12)	C5—C6—C7—C2	−1.8 (2)
O5^i^—Cd1—N1—C8	−65.15 (12)	C10—C9—C8—N1	−0.2 (2)
O5—Cd1—N1—C12	−62.64 (12)	C13—C9—C8—N1	177.96 (14)
O5^i^—Cd1—N1—C12	117.36 (12)	C8—C9—C13—O4	2.5 (2)
O7—Cd2—O6—C14	4.53 (9)	C8—C9—C13—N2	−175.32 (14)
O9—Cd2—O6—C14	39.56 (11)	C10—C9—C13—O4	−179.50 (15)
O13—Cd2—O6—C14	112.11 (10)	C10—C9—C13—N2	2.7 (2)
O14—Cd2—O6—C14	−71.89 (10)	C11—C10—C9—C8	−0.3 (2)
N3—Cd2—O6—C14	−155.46 (10)	C11—C10—C9—C13	−178.28 (15)
O6—Cd2—O7—C14	−4.58 (9)	C12—C11—C10—C9	0.2 (2)
O9—Cd2—O7—C14	−158.73 (10)	N1—C12—C11—C10	0.3 (2)
O13—Cd2—O7—C14	−77.43 (10)	O6—C14—C15—C16	1.9 (2)
O14—Cd2—O7—C14	102.97 (10)	O6—C14—C15—C20	−175.00 (15)
N3—Cd2—O7—C14	24.42 (13)	O7—C14—C15—C16	−179.77 (15)
O6—Cd2—O9—C21	159.44 (9)	O7—C14—C15—C20	3.3 (2)
O7—Cd2—O9—C21	−172.93 (10)	C14—C15—C16—O8	4.0 (2)
O13—Cd2—O9—C21	87.11 (10)	C14—C15—C16—C17	−176.23 (15)
O14—Cd2—O9—C21	−87.45 (10)	C20—C15—C16—O8	−179.05 (15)
N3—Cd2—O9—C21	3.57 (13)	C20—C15—C16—C17	0.7 (2)
O6—Cd2—N3—C28	10.89 (11)	C14—C15—C20—C19	176.50 (16)
O6—Cd2—N3—C32	−171.74 (12)	C16—C15—C20—C19	−0.5 (2)
O7—Cd2—N3—C28	−12.09 (15)	O8—C16—C17—C18	179.66 (16)
O7—Cd2—N3—C32	165.28 (11)	C15—C16—C17—C18	−0.1 (3)
O9—Cd2—N3—C28	172.86 (10)	C16—C17—C18—C19	−0.7 (3)
O9—Cd2—N3—C32	−9.77 (16)	C17—C18—C19—C20	1.0 (3)
O13—Cd2—N3—C28	92.64 (12)	C18—C19—C20—C15	−0.4 (3)
O13—Cd2—N3—C32	−90.00 (12)	O9—C21—C22—C23	−3.4 (2)
O14—Cd2—N3—C28	−91.38 (12)	O9—C21—C22—C27	179.68 (14)
O14—Cd2—N3—C32	85.99 (12)	O10—C21—C22—C23	176.13 (14)
Cd1—O2—C1—O1	−21.6 (2)	O10—C21—C22—C27	−0.8 (2)
Cd1—O2—C1—C2	154.63 (10)	C21—C22—C23—O11	2.0 (2)
Cd2—O6—C14—O7	−7.63 (15)	C21—C22—C23—C24	−176.99 (14)
Cd2—O6—C14—C15	170.65 (14)	C27—C22—C23—O11	178.95 (14)
Cd2—O7—C14—O6	8.74 (17)	C27—C22—C23—C24	0.0 (2)
Cd2—O7—C14—C15	−169.53 (12)	C21—C22—C27—C26	177.76 (15)
Cd2—O9—C21—O10	−2.86 (17)	C23—C22—C27—C26	0.8 (2)
Cd2—O9—C21—C22	176.66 (11)	O11—C23—C24—C25	−179.63 (15)
Cd1—N1—C8—C9	−176.84 (11)	C22—C23—C24—C25	−0.6 (2)
C12—N1—C8—C9	0.7 (2)	C23—C24—C25—C26	0.5 (3)
Cd1—N1—C12—C11	176.90 (12)	C24—C25—C26—C27	0.2 (3)
C8—N1—C12—C11	−0.7 (2)	C25—C26—C27—C22	−0.9 (2)
Cd2—N3—C28—C29	177.67 (12)	N3—C28—C29—C30	−0.3 (2)
C32—N3—C28—C29	0.2 (2)	N3—C28—C29—C33	178.28 (14)
Cd2—N3—C32—C31	−177.42 (12)	C28—C29—C30—C31	0.4 (2)
C28—N3—C32—C31	0.0 (2)	C33—C29—C30—C31	−178.11 (15)
O1—C1—C2—C3	163.89 (15)	C28—C29—C33—O12	5.2 (2)
O1—C1—C2—C7	−11.0 (2)	C28—C29—C33—N4	−173.41 (15)
O2—C1—C2—C3	−12.6 (2)	C30—C29—C33—O12	−176.29 (16)
O2—C1—C2—C7	172.54 (14)	C30—C29—C33—N4	5.1 (2)
C1—C2—C3—O3	8.9 (2)	C29—C30—C31—C32	−0.2 (2)
C1—C2—C3—C4	−169.65 (14)	C30—C31—C32—N3	0.1 (2)

Symmetry code: (i) −*x*, −*y*, −*z*+1.

### Hydrogen-bond geometry (Å, º)

Cg1 is the centroid of the C2–C7 ring.

**Table:**

*D*—H···*A*	*D*—H	H···*A*	*D*···*A*	*D*—H···*A*
N2—H2*A*···O11^ii^	0.88	2.21	3.025 (2)	154
N2—H2*B*···O1^iii^	0.88	2.23	3.054 (2)	156
N4—H4*B*···O13^iv^	0.88	2.13	2.937 (2)	151
O3—H3···O2	0.84	1.81	2.548 (2)	146
O5—H51···O7^v^	0.78 (3)	1.95 (3)	2.722 (2)	172 (3)
O5—H52···O1^i^	0.82 (3)	1.89 (3)	2.687 (2)	165 (3)
O8—H81···O6	0.84	1.83	2.569 (2)	146
O11—H111···O5^vi^	0.84	2.52	3.048 (2)	122
O11—H111···O9	0.84	1.79	2.535 (2)	146
O13—H131···O3^vi^	0.76 (3)	2.02 (3)	2.760 (2)	165 (2)
O13—H132···O4^vii^	0.79 (3)	1.88 (3)	2.656 (2)	168 (3)
O14—H141···O15^iii^	0.78 (3)	1.92 (3)	2.693 (2)	178.1 (5)
O14—H142···O10^viii^	0.84 (3)	1.89 (3)	2.720 (2)	178 (4)
O15—H15*A*···O16*A*	0.86 (2)	1.95 (2)	2.764 (4)	156 (2)
O15—H15*A*···O16*B*	0.86 (2)	1.93 (2)	2.689 (5)	146 (2)
O15—H15*B*···O12	0.84 (3)	2.08 (3)	2.880 (2)	159 (3)
O16*A*—H161···O8^vii^	0.83 (5)	2.53 (5)	3.139 (4)	132 (4)
O16*A*—H162···O1^ix^	0.89 (4)	2.14 (3)	2.965 (4)	153 (5)
O16*B*—H164···O8	0.91 (2)	1.91 (2)	2.748 (2)	153 (3)
C28—H28···O6	0.95	2.35	3.101 (2)	136
N4—H4*A*···*Cg*1	0.88	2.69	3.470 (2)	148

Symmetry codes: (i) −*x*, −*y*, −*z*+1; (ii) −*x*+2, −*y*+1, −*z*+1; (iii) *x*+1, *y*, *z*; (iv) −*x*+1, −*y*+1, −*z*+2; (v) *x*−1, *y*−1, *z*; (vi) *x*+1, *y*+1, *z*; (vii) −*x*+1, −*y*+1, −*z*+1; (viii) −*x*+2, −*y*+1, −*z*+2; (ix) −*x*, −*y*+1, −*z*+1.
